# The control of translational accuracy is a determinant of healthy ageing in yeast

**DOI:** 10.1098/rsob.160291

**Published:** 2017-01-18

**Authors:** Tobias von der Haar, Jane E. Leadsham, Aimie Sauvadet, Daniel Tarrant, Ilectra S. Adam, Kofo Saromi, Peter Laun, Mark Rinnerthaler, Hannelore Breitenbach-Koller, Michael Breitenbach, Mick F. Tuite, Campbell W. Gourlay

**Affiliations:** 1 Kent Fungal Group, School of Biosciences, University of Kent, Canterbury CT2 7NJ, UK; 2 Department of Cell Biology, University of Salzburg, Hellbrunnerstrasser 34, 5020 Salzburg, Austria

**Keywords:** protein synthesis, translational accuracy, ageing, chaperones, homeostasis

## Abstract

Life requires the maintenance of molecular function in the face of stochastic processes that tend to adversely affect macromolecular integrity. This is particularly relevant during ageing, as many cellular functions decline with age, including growth, mitochondrial function and energy metabolism. Protein synthesis must deliver functional proteins at all times, implying that the effects of protein synthesis errors like amino acid misincorporation and stop-codon read-through must be minimized during ageing. Here we show that loss of translational accuracy accelerates the loss of viability in stationary phase yeast. Since reduced translational accuracy also reduces the folding competence of at least some proteins, we hypothesize that negative interactions between translational errors and age-related protein damage together overwhelm the cellular chaperone network. We further show that multiple cellular signalling networks control basal error rates in yeast cells, including a ROS signal controlled by mitochondrial activity, and the Ras pathway. Together, our findings indicate that signalling pathways regulating growth, protein homeostasis and energy metabolism may jointly safeguard accurate protein synthesis during healthy ageing.

## 1. Introduction

Textbook descriptions of the genetic code depict a static information transfer system in which codons encode a single amino acid. In reality, however, the power of the translational machinery to distinguish correct amino acids from incorrect ones is not absolute, and errors in the decoding process can, therefore, occur. Error levels are generally low with measured amino acid misincorporation frequencies in eukaryotes of 10^−3^ to 10^−6^, depending on the organism and codon in question [1–4]. Other types of translational error, such as stop-codon read-through or ribosomal frame-shifting, occur at similarly low levels (typically much below 1%).

Although error levels are low, they are not negligible: studies on sequence evolution have revealed that organisms prefer less error-prone codons at structurally sensitive sites [5,6], suggesting that natural amino acid misincorporation levels affect protein function sufficiently to allow evolutionary selection. In this context, it is worth noting that error frequencies cited in the literature refer to incorporation of a single non-cognate amino acid at individual codons, but it is currently unknown how high total misincorporation levels of all 19 non-cognate amino acids are for any codon.

Translational errors are relatively well understood in terms of their biophysical origin. Two important mechanisms leading to amino acid misincorporation are the catalysis of peptidyl-transfer by the ribosome even though the anticodon of the A-site tRNA does not fully match the codon (codon misreading), and the charging of tRNAs with inappropriate amino acids (misacylation). The biochemistry of misincorporation at both levels has been studied in detail (e.g. [7,8]), and these data allow a limited prediction of types and rates of errors occurring on specific codons *in vivo* [3,9].

Translational errors generally have negative consequences for the cell. Random amino acid substitutions in proteins have a wide variety of effects depending on the site and type of substitution, but the average outcome of such substitutions is a loss of function [10]. Error levels observed under physiological conditions are generally compatible with protein function, but at structurally particularly sensitive sites of some proteins only the least error-prone codons appear to result in appropriate protein activity [5,6]. Error levels that are increased beyond the normal physiological range cause proteotoxic stress [11] and induce stress responses [12], although they can be tolerated and even adapted to in baker's yeast. However, such adaptation diverts energy and comes at the cost of other evolutionary trade-offs [13].

Despite the generally negative consequences of translational errors, evolution has exploited specific types of error and incorporated them into biological pathways. For example, in *Candida albicans*, stochastic decoding of CUG as either serine or leucine increases phenotypic diversity [14].

Despite our good understanding of the biochemical sources of errors, we know little about their physiological regulation. Anecdotal evidence suggests that error levels are not static but respond to regulatory input from signalling pathways [15,16]. This suggests that cells can actively manage error levels depending on requirements. Here, we ask how translational errors interact with the particular demands on protein quality in ageing baker's yeast. We uncover evidence for a complex regulatory programme that controls translational errors, thereby protecting proteome integrity and cell viability during early ageing.

## 2. Results

### 2.1. High-level translational errors are incompatible with healthy ageing

**Figure 1.**
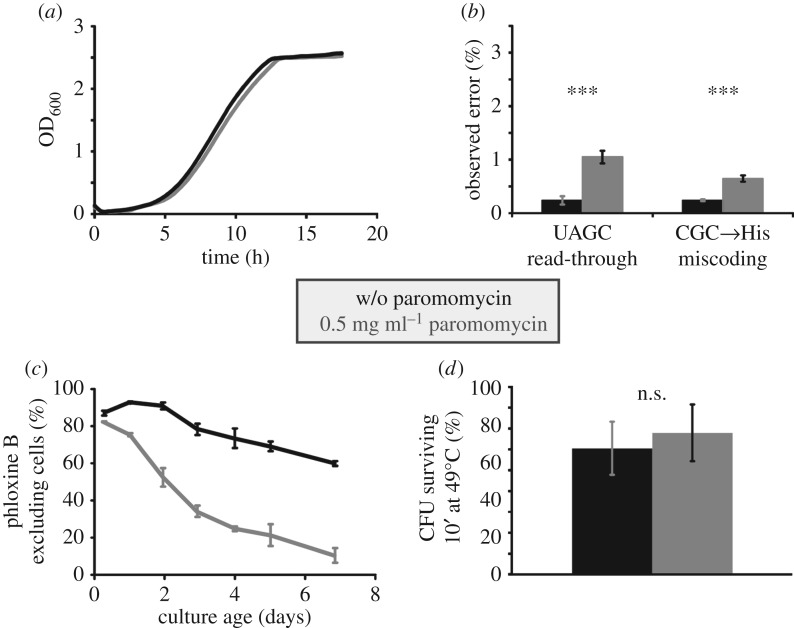
Paromomycin-induced translational errors accelerate loss of viability during ageing. All panels show data for a laboratory yeast strain (BY4741) transformed to Ura^+^ and grown in SC−Ura medium, with or without 0.5 mg ml^−1^ paromomycin (data in grey and black, respectively). (*a*) At this paromomycin concentration, the logarithmic growth rate is reduced by less than 5%. Curves shown represent typical replicates from four independently grown transformants. (*b*) Under paromomycin treatment, stop-codon read-through is increased fivefold and amino acid misincorporation threefold compared with non-treated cells. Bars indicate average and standard deviation for eight separate transformants. (*c*) Survival of cells in culture was assessed by determining the percentage of cells able to exclude phloxine B. Paromomycin-treated cells lose viability faster than non-treated cells. Shown are averaged data obtained with three separate transformants for each condition. (*d*) Paromomycin treatment does not significantly affect survival of a heat stress. Bars represent average and standard deviation from three separate transformants. In all panels, statistical significance as determined by one-way ANOVA and *post hoc* testing (Tukey's HSD) is indicated as follows: n.s., *p* > 0.05; *** *p* < 0.001.

In order to investigate the effect of translational errors on the rate of ageing, we initially investigated the effect of the error-inducing drug paromomycin. In initial pilot experiments, we selected a working concentration at which the logarithmic growth rate is reduced by less than 5% (figure 1*a*), which we reasoned would be sufficient to elicit observable phenotypes, but would avoid approaching non-physiological levels of errors that would simply kill the cells. Using an established dual luciferase reporter system for measuring stop-codon read-through and amino acid misincorporation [2], we determined that at this concentration, stop-codon read-through on UAGC stop codons is increased about fivefold, whereas amino acid misincorporation is increased about threefold (figure 1*b*).

Despite the small effect of paromomycin at this concentration on growth rates, we observed a strong effect on chronological lifespan in wild-type yeast cells (figure 1*c*). In these assays, we distinguished live and dead cells via the ability of live cells to exclude the stain phloxine B, which stains dead cells bright pink [17]. With strain BY4741 grown in −Ura dropout medium, the proportion of phloxine B excluding cells dropped over time with an average half-life of 8–10 days. In the presence of the drug, this was significantly shortened to a half-life of 2–3 days. Interestingly, we observed that paromomycin at this concentration did not affect the cells' ability to survive heat shock, a condition that also leads to transient denaturation and aggregation of proteins (figure 1*d*), as survival of the cells following a 10-min incubation at 49°C was not significantly affected. Thus, an increase in translation error induced by paromomycin treatment has a specific and strong effect on yeast ageing that is separable from its effects on growth rate and the heat-shock response.

**Figure 2.**
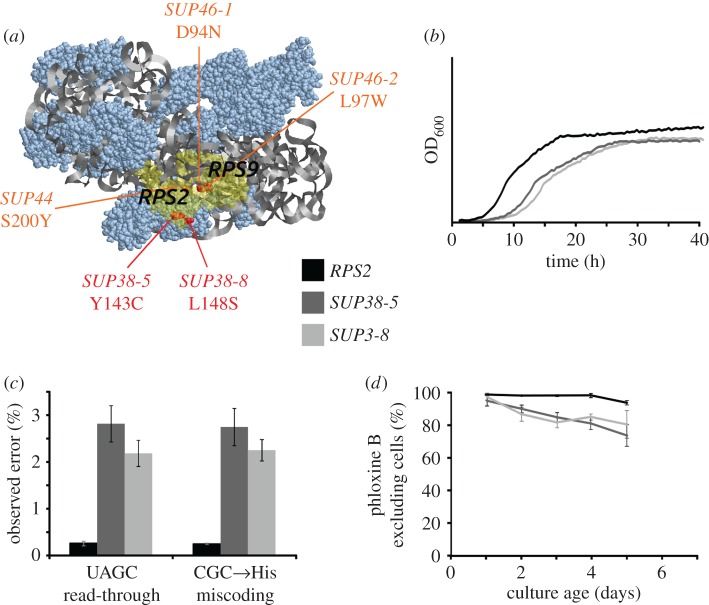
Reduced accuracy mutants accelerate loss of viability during ageing. Data were generated with an *RPS2* shuffling strain derived from W303 [15], containing a chromosomal deletion of the *RPS2* gene and plasmid-borne copies of either wild-type (black data) or mutant (grey data) copies of the *RPS2* gene. (*a*) Location of the *SUP38-5* and *SUP38-8* mutations in comparison with other known accuracy mutations in the yeast small ribosomal subunit. (*b*) Growth rates of *RPS2* mutant and wild-type strains in SC−Ura medium. Curves shown represent typical replicates from four independently grown transformants. (*c*) The *SUP38* mutations strongly increase decoding errors as measured with the dual luciferase reporter system. Bars indicate average and standard deviation for five separate transformants. (*d*) Cells containing *RPS2* mutant genes lose viability faster than cells containing the wild-type gene. Shown are averaged data obtained with three separate transformants for each condition.

To confirm that the faster ageing phenotype was caused by effects of translational errors, we repeated these experiments with another error-inducing drug, nourseothricin, with similar results (data not shown). In addition, we also repeated the experiments with genetic modifiers of error levels. For this work, we used several *SUP38* mutants, obtained from the Yeast Genetic Resource Centre (Japan), which in the literature had been described as allelic to *SUP44/RPS2* [18]. When we amplified the *RPS2* gene of the respective strains by PCR and sequenced the recovered DNA, we observed that the *SUP38-5* mutant encoded a Y143C variant of the Rps2 protein, whereas the *SUP38-8* and *SUP38-9* mutants encoded an L148S variant. Interestingly, in the structure of RPS2 these two mutations are located on the opposite face of the site of other known *SUP44* and *SUP46 (RPS9)* accuracy mutants, which are all situated near the Rps2/Rps9 interface (figure 2*a*).

In order to quantify the effect of these mutants on translational accuracy, we constructed plasmids containing the wild-type and *SUP38* alleles of *RPS2* including the natural *RPS2* regulatory sequences, and then introduced these plasmids into a previously described *RPS2* shuffling strain [15]. Strains containing the *SUP38* mutants as the sole source of Rps2 showed a significantly reduced growth rate (72% and 61% of the wt growth rate for *SUP38-5* and *SUP38-8*, respectively), as well as a lower final biomass and longer lag phase (figure 2*b*), and strongly increased levels of stop-codon read-through and amino acid misincorporation (figure 2*c*). Both mutants also affected viability following the logarithmic growth phase, with linear losses in viability from day 1, whereas the strain containing wild-type Rps2 only showed notable loss in viability from day 4 onwards (figure 2*d*).

Interestingly, although the *rps2* mutants affected accuracy and growth more strongly than the paromomycin treatment, the mutants affected ageing quantitatively less strongly. A number of issues may have contributed to this apparent discrepancy. For each of the 61 sense codons, there are 19 possibilities of incorporating amino acids, and the luciferase reporter systems only allow us to evaluate two of these more than 1000 error combinations. Paromomycin and *rps2* mutations may affect particular translational errors in different ways and the *average* error induced by these treatments may differ from the error reported by the luciferase constructs. Moreover, the *RPS2* shuffling strain was constructed in a different strain background (W303) from the strain used in the paromomycin experiments (BY4741). The two strains show distinct differences in their ageing behaviour, which may further explain why the levels of observed errors and the effect on ageing do not correlate quantitatively. Other explanations for the lack of correlation may include pleiotropic effects of error-inducing drugs, which exacerbate the ageing effect beyond that expected purely from the translational errors induced by them.

Recently, deletion of an rRNA cytosine methyltransferase (NSUN5/*RCM1*) was shown to reduce translational accuracy yet increase lifespan in yeast, worms and flies [19]. Because in that work stop-codon read-through was the only translational error assessed, we assayed amino acid misincorporation levels in the *rcm1* deletion strain with our suite of dual luciferase reporters (electronic supplementary material, figure S1). The results show that translational accuracy in the *rcm1* deletion strain is generally higher than wild-type under the conditions we used, and is moreover selective as the *rcm1* strain does not show any changes in accuracy for the AGG→Lys miscoding event. It is possible that differences in levels and types of translational inaccuracy between the *rcm1* deletion and our conditions cause the differential effects on lifespan and stress responses. However, as a minimum interpretation of our results, we conclude that significant levels of general amino acid misincorporation interfere with healthy ageing, in multiple strain backgrounds. These results with translational errors mirror the results obtained by others for transcriptional errors [20].

### 2.2. Age-dependent defects in protein folding are exacerbated by translational inaccuracy

**Figure 3.**
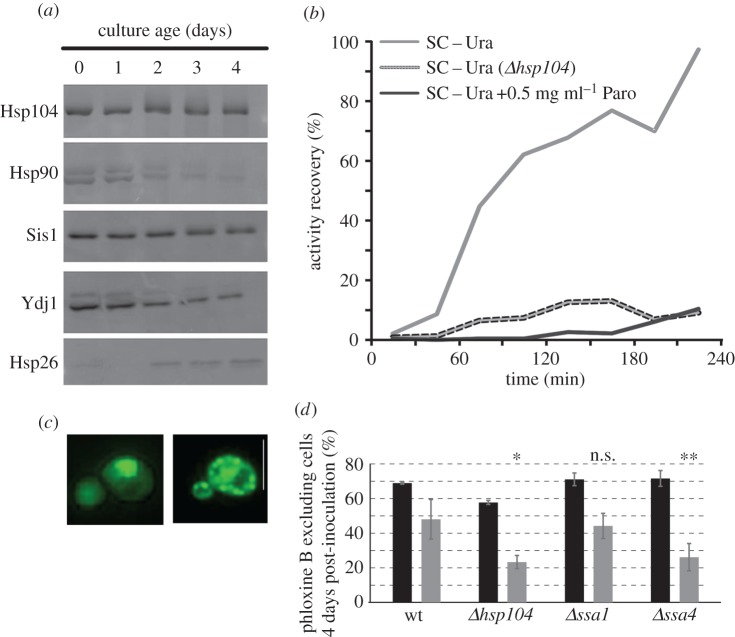
Translational accuracy interacts with cell ageing via proteotoxicity effects. (*a*) Regulation of the yeast chaperone system during cell ageing. Hsp104, the Hsp40 Sis1 and Hsp26 remain expressed or are upregulated in ageing cells. Hsp90 and the Hsp40 Ydj1 show expression patterns typical for most cytoplasmic proteins, which decline during ageing. (*b*) Chaperone support and high translational fidelity are both required to retain the folding competence of difficult-to-fold proteins. The panel shows the results of luciferase refolding experiments, which involve expression of a bacterial luxAB luciferase in yeast, unfolding of the luciferase via heat shock and observation of the refolding in the presence of the translation inhibitor cycloheximide. Deletion of Hsp104 or reduction of translational fidelity by inclusion of paromomycin (Paro) in the growth medium both prevent refolding-dependent recovery of the luciferase activity. The experiment shown was independently reproduced twice, with similar results. (*c*) Paromomycin-treated cells expressing GFP-tagged Hsp104 display numerous GFP-decorated aggregates indicative of widespread protein misfolding (right). An untreated cell is show for comparison (left). Size bar = 10 µm. (*d*) Paromomycin treatment and chaperone deletions show chemicogenetic interactions. The viability of strains 4 days post-inoculation was determined in the absence (black bars) or presence (grey bars) of 0.5 mg ml^−1^ paromomycin. Bars indicate averages and standard deviations for three separate transformants. Significance of the interaction is indicated as determined by two-way ANOVA and *post hoc* testing (Tukey's HSD). n.s., not significant; *p < 0.05; **p < 0.01.

The observed negative effects of reduced translational accuracy on ageing are consistent with our general knowledge on the interaction between translational fidelity and protein function. Random misincorporation of amino acids into proteins is known to result in a net reduction of folding competence [10]. The ageing proteome requires substantial assistance from molecular chaperones, some of which are upregulated in ageing cells (figure 3*a*). As the cellular proteome adapts to stationary phase in yeast, levels of most proteins in the cell decline as exemplified by Hsp90 and Ydj1 in figure 3*a*. By contrast, chaperones like Hsp104 and Sis1 remain highly expressed, and specialized chaperones like Hsp26 become substantially upregulated with age. These changes in chaperone expression are thought to provide a more folding-supportive environment which is crucial for supporting the function of difficult-to-fold proteins. This is directly illustrated in the classic luciferase refolding assay [21], which assesses the refolding of a heat-denatured bacterial luciferase following thermal unfolding in the absence of protein synthesis (achieved by addition of cycloheximide). Refolding is observed in wild-type cells, but is fully abrogated in the absence of Hsp104, as previously reported (figure 3*b*). Besides chaperone assistance, physiological levels of translational fidelity are also crucial for protein re-folding, as the application of paromomycin at the same concentration as used for experiments in figure 1 prevents luciferase refolding even in the presence of Hsp104 (figure 3*b*, black solid line).

Besides interfering with the refolding of a model protein, paromomycin also generates more general problems with proteome integrity. In a strain in which Hsp104 is GFP-tagged, application of paromomycin produces GFP-decorated aggregates consistent with the appearance of more widespread protein aggregation (figure 3*c*). Together, these results demonstrate that drug-induced translational errors impair the folding competence of the proteome, and that this challenges the same molecular chaperone network that is also upregulated during ageing.

To provide direct proof for an interaction between translational errors and the chaperone system during ageing, we assessed yeast ageing under the combined treatment of chaperone deletion and application of paromomycin (figure 3*d*). In an *hsp104* deletion strain, we did indeed observe that the viability 4 days post inoculation is reduced compared to the effect of paromomycin on a wild-type strain. Moreover, we observed similar interactions with Hsp70 family members and these were surprisingly isoform-specific, with deletion of the *SSA4* gene, but not of the *SSA1* gene, producing a highly significant interaction with paromomycin treatment (*p* *=* 0.96 for *ssa1, p* = 0.005 for *ssa4*).

In summary, we demonstrate that chaperone levels are upregulated during cellular ageing, that translational inaccuracy interferes with chaperone-dependent protein folding and that impairment of chaperones and reduced translational accuracy interact genetically to reduce fitness during ageing. Both ageing and translational inaccuracy appear to reduce the folding competence of the proteome, and require increased reliance on specialized chaperone networks to protect proteome integrity. The combined effect of translational errors and age-related problems with protein folding potentially may overwhelm the chaperone machinery, which provides a rationale for the reduced viability under high error conditions during ageing.

### 2.3. The effect of ageing-dependent physiological parameters on translational accuracy

**Figure 4.**
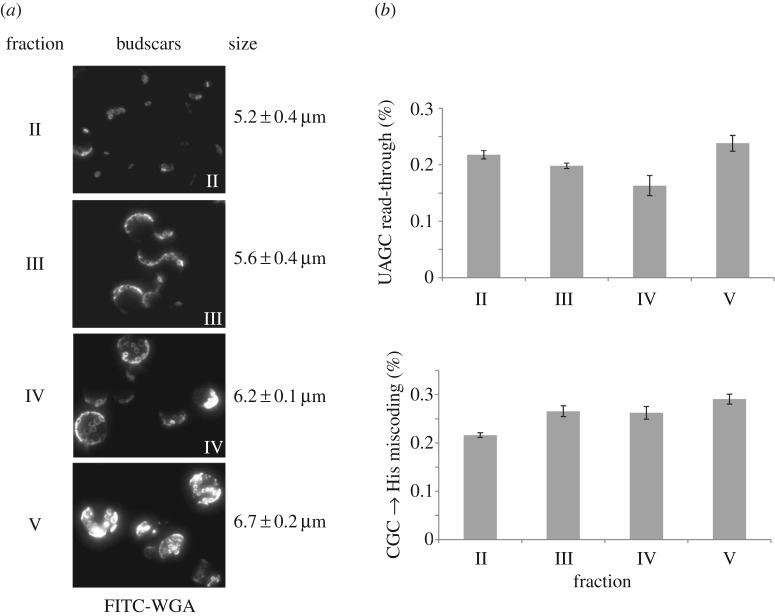
Translational accuracy does not change significantly during ageing. Yeast cells were separated by centrifugal counterflow elutriation. (*a*) Staining of bud scars using FITC-wheat germ agglutinin and determination of average size demonstrates successful separation into fractions of distinct age classes. (*b*) Dual luciferase reporter measurements reveal no significant changes in stop-codon read-through or in misincorporation of histidine on a CGC (arginine) codon. Bars indicate average and standard deviation of three technical replicates of one elutriation experiment.

A substantial number of physiological parameters are known to change during ageing; however, all data available to date indicate that translational accuracy itself remains relatively constant with age in all investigated systems [22–25]. The available data are limited by the fact that all previous investigations into ageing and translational accuracy focused on the chronological mode of cell ageing. Since gene deletions affecting chronological and replicative ageing show little overlap [26], we reasoned that accuracy effects might also differ between these two modes of ageing, and therefore measured accuracy for the first time in replicatively ageing cells. To do this, we separated cells transformed with the dual luciferase reporters of different replicative ages via centrifugal elutriation [27], and then conducted luciferase assays on the different fractions. Consistent with all previous studies on chronological ageing, we did not observe significant changes in translational accuracy in cells of different replicative age (figure 4). These findings are intriguing because many individual processes known to be affected by ageing have been linked to altered translational accuracy. *How* accuracy is maintained in the face of widespread changes to cellular physiology is thus not understood.

One particularly intriguing question concerns the interplay between translational accuracy and translational speed. About half of the demand on the translational machinery in yeast growing exponentially in rich medium is required to produce protein for growth [28]. Ageing cells in stationary phase do not have this requirement, and together with the generally reduced protein content of such cells, the demand on the translational machinery should be a fraction of that required during logarithmic growth.

An intuitive assumption found in several places in the literature is that slower ribosomes translate more accurately, and one study produced experimental evidence for this hypothesis [29]. However, older experimental studies based on the incorporation of leucine during translation of poly(U) RNA *in vitro* found the opposite relationship, i.e. the translational machinery is more accurate the more active it is (summarized in [30]).

**Figure 5.**
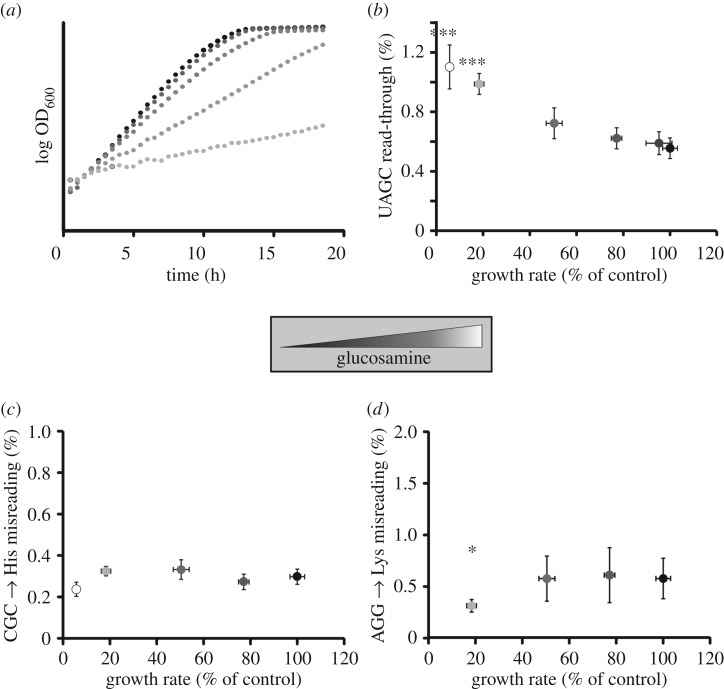
The interaction between growth rate and translational accuracy. (*a*) Growth rate was controlled by adding increasing concentrations of glucosamine to standard medium containing 2% glucose as carbon source. Curves shown are typical for curves generated with three separate transformants. (*b*–*d*) Dual luciferase reporter measurements reveal changes in accuracy correlated with growth rates. Stop-codon read-through (*b*) shows a trend to increase with reduced growth rate. By contrast, amino acid misincorporation (*c*,*d*) remains constant over a wide growth-rate range, but decreases at very low growth rates. Statistical significance of difference in error levels to no glucosamine treatment, as determined by ANOVA and *post hoc* testing, is indicated (*p < 0.05; ***p < 0.001). (*b*–*d*) Outlier data were identified based on Cook's distance (more than threefold difference to mean) and removed from the calculations. In all panels, error bars indicate the standard deviation based on at least three separate transformants.

In order to investigate the relationship between growth rates, translational activity and translational speed, we initially used a competitive inhibitor of glycolysis. Glucosamine can be used to control logarithmic growth rates of yeast over a wide range [31] (figure 5*a*). When we applied the luciferase reporter measurements to cells grown with varying concentrations of glucosamine, we observed that stop-codon read-through on *UAGC* stop codons displayed a strong negative correlation with growth rates (figure 5*b*), increasing about twofold at the lowest growth rates measured (which are approximately 10-fold slower than growth rates in standard medium). By contrast, amino acid misincorporation as measured using two distinct reporters remained constant over a wide range of growth rates (figure 5*c,d*). At the very lowest growth rates, we observed a decrease in misincorporation rates with both reporters, consistent with increased accuracy at such low growth speeds.

**Figure 6.**
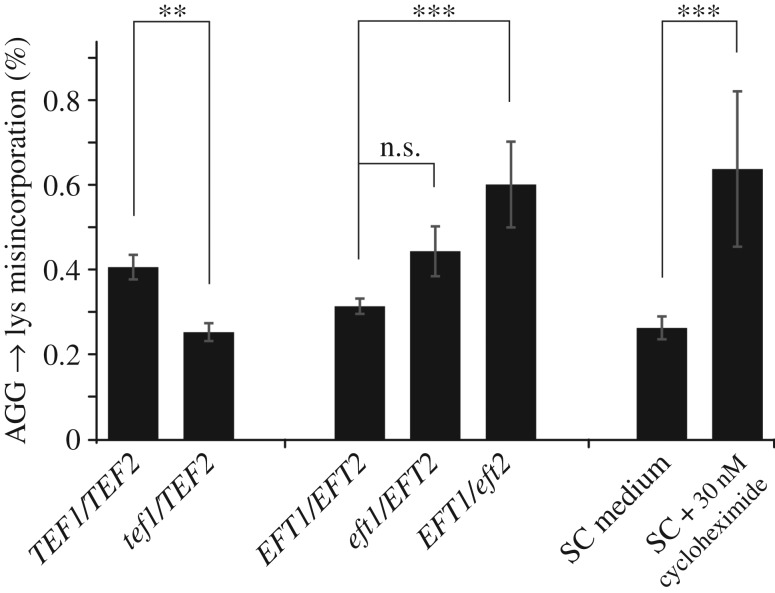
The interaction between translational activity and translational accuracy. Deletion of the *TEF1* gene, one of two identical genes encoding elongation factor 1A in yeast, reduces lysine misincorporation on an AGG codon. By contrast, deletion of either of the two identical genes encoding elongation factor 2 increase the same error. Application of low concentrations of cycloheximide, which also blocks the translocation step of translation elongation, has a similar effect as eEF2 depletion. Bars indicate averages and standard deviations for six separate transformants. Statistical significance as determined by one-way ANOVA and *post hoc* testing (Tukey's HSD) is indicated as follows: n.s., *p* > 0.05; **p < 0.01; ***p < 0.001.

Next, we directly manipulated translational speed by either altering the levels of translation elongation factors, or by applying drugs that interfere with efficient translation elongation. In a *Δtef1/TEF2* yeast strain where one of the two identical genes encoding elongation factor 1A has been deleted, and where Tef1 levels are reduced by 40% (data not shown), we observe lower levels of amino acid misincorporation (figure 6). By contrast, in an *EFT1/Δeft2* strain where levels of elongation factor 2 are lower and the speed of translocation is therefore reduced, we observed a significant decrease in the accuracy of translation and higher levels of misincorporation. Application of cycloheximide at a concentration that leads to a 20% reduction in growth, and which mimics eEF2 depletion as cycloheximide is also a translocation inhibitor, had a similar effect as reductions in eEF2 content.

Overall, our experiments paint a varied picture of the connection between translational speed and translational accuracy, but they do not reveal a clear correlation between the two. Very low growth rates appear to reduce amino acid misincorporation, although the effect was overall of borderline significance with *p* = 0.20 for His misincorporation on CGC codons and *p* = 0.02 for Lys misincorporation on AGG. This could indicate that at such low or zero growth rates, cellular signals operate that reduce translational errors, and that such signals trigger modifications in ribosomal function that can counteract the reduced efficiency one might expect to find in an ageing translational machinery.

### 2.4. Signalling pathways that impinge on translational accuracy

**Figure 7.**
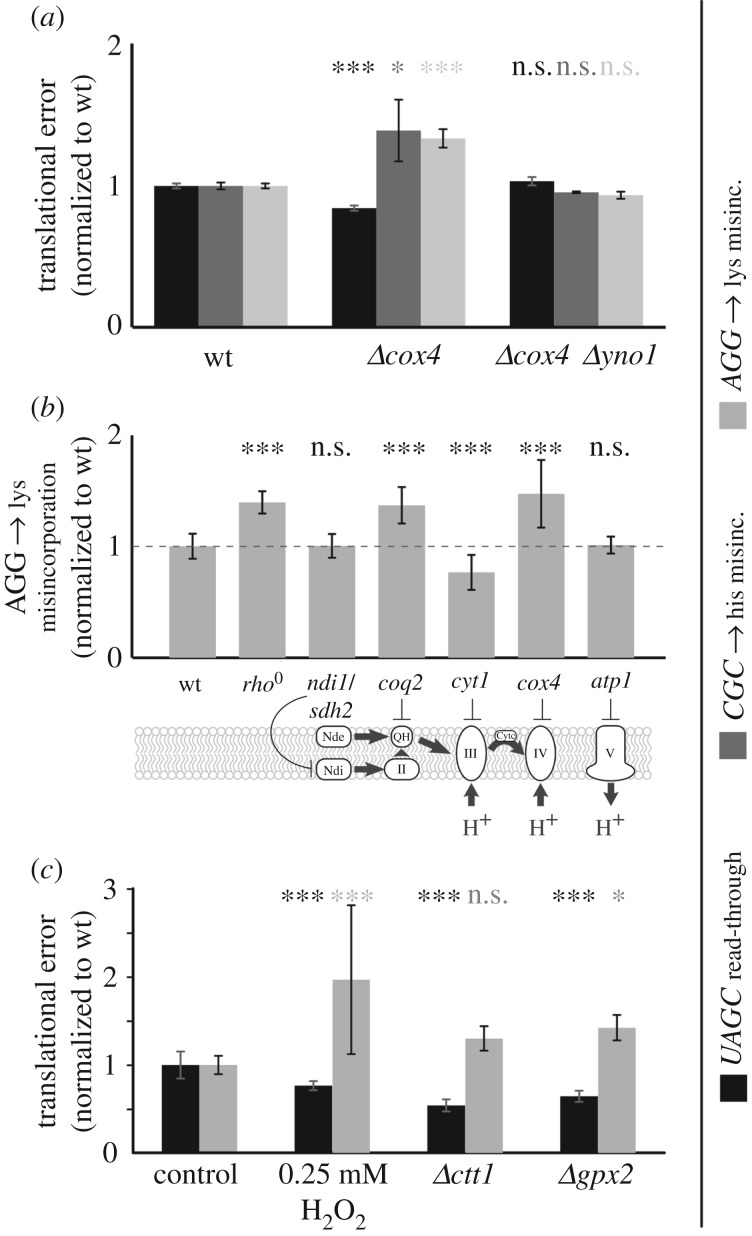
A mitochondria-dependent ROS signal controls translational accuracy. (*a*) Deletion of *cox4*, a nuclear encoded subunit of the mitochondrial complex IV, increases amino acid misincorporation but slightly decreases stop-codon read-through. This effect is rescued in a *cox4 yno1* double mutant, in which the accumulation of ROS resulting from the *cox4* deletion is prevented. (*b*) Impaired function of complex II (*coq2*), as well as removal of mitochondrial DNA (*rho*^0^), produces an increase in amino acid misincorporation similar to *cox4* deletion. (*c*) Increases in intracellular ROS concentration by external application of H_2_O_2_, or by deletion of genes that encode ROS-removing activities, produce similar effects as deletion of *cox4*. Bars indicate averages and standard deviations for eight (*a*,*b*) or six (*c*) independent transformants. Statistical significance as determined by one-way ANOVA and *post hoc* analyses (Tukey's HSD) are indicated as follows: n.s., *p* > 0.05; *p < 0.05; ***p < 0.001.

Because one of the hallmarks of ageing is a reduced efficiency in mitochondrial function, we assessed translational accuracy under conditions of impaired mitochondrial function. Our initial model for mitochondrial dysfunction was a deletion of the *COX4* gene, which encodes a central subunit of the mitochondrial complex IV. Upon *cox4* deletion, we observed a small but significant increase in the levels of amino acid misincoporation as measured using two different reporter constructs, as well as a small but significant decrease in the levels of read-through on a UAGC stop codon (figure 7*a*). By contrast, read-through of a UGAC stop codon was not significantly affected (data not shown). We tested other mitochondrial defects to see whether they would reproduce the observed decrease in amino acid misincorporation levels (figure 7*b*), and found that this is a general phenomenon which occurs in many (though not all) mutants with defects in the mitochondrial electron transport chain.

One of the effects of a reduction in mitochondrial efficiency is the production of ROS, via a signalling pathway that involves the ER-localized Yno1 NADPH oxidase. We previously showed that deletion of *yno1* abrogates the production of ROS upon deletion of *cox4* [32]. In a *cox4 yno1* double deletion strain, we observed no significant changes in either stop-codon read-through or amino acid misincorporation (figure 7*a*), which indicates that the changes in translational accuracy are dependent on the altered ROS levels in *cox4* strains. Consistent with this notion, the direct application of low levels of ROS, or raising intracellular ROS levels by deleting genes involved in the removal of ROS from the cell, produced identical patterns of increased amino acid misincoporation and decreased UAGC read-through as deletion of the *COX4* gene (figure 7*c*).

**Figure 8.**
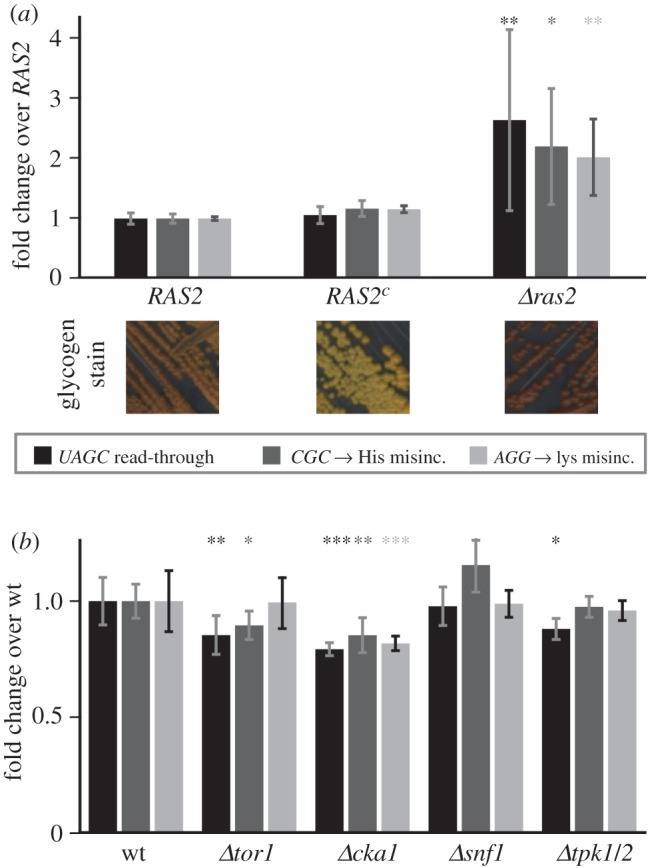
Signalling pathways impinge on levels of translational accuracy. (*a*) Deletion of the *RAS2* gene significantly decreases translational accuracy, whereas introduction of the Val19 mutation which constitutively activates Ras2 (*RAS2^c^*) does not significantly affect accuracy. Glycogen stains below the graphs verify phenotypes expected for activity changes in the Ras signalling pathway. (*b*) Knockouts of genes acting in other signalling pathways tested affect translational fidelity with lower or no statistical significance. Bars indicate average and standard deviation from at least six independent transformants. Statistical significance as tested by Kruskal–Wallis test (*a*) or ANOVA (*b*) and *post hoc* testing (Tukey's HSD) is indicated as follows: *p < 0.05; **p < 0.01; ***p < 0.001.

Since reduced mitochondrial activity signals to decrease translational accuracy, but ageing cells seem able to maintain levels of protein synthesis accuracy despite reduced mitochondrial function, we reasoned that cells might use other signalling pathways to counteract the mitochondrial signal. We therefore surveyed the main signalling pathways for their effects on translational accuracy. We observed that a deletion of the *RAS2* gene led to a distinctive phenotype in which a subset of colonies showed substantially (threefold to fourfold) increased levels of expression of all our error reporters, whereas other colonies showed only slightly increased levels. This pattern was highly reproducible in three independent experiments conducted with independently transformed samples of *ras2* deletions in different strain backgrounds. In terms of the experimental average, this effect appears as a two to threefold increase in the average reporter activity as well as a high standard deviation (figure 8). We assessed significance of these results using the non-parametric Kruskal–Wallis rank sum test (for the experiment shown in figure 8*a*, ANOVA *p* = 3.6 × 10^−5^, Kruskal–Wallis *p* = 1.0 × 10^−6^). Post hoc analyses following the Kruskal–Wallis test indicate significant differences in the *ras2* deletion mutant for all three error reporters (figure 8*a*).

In contrast with the *ras2* deletion, a constitutively active Val19 mutant maintained error levels that were indistinguishable from wild-type levels. Together, these results indicate that in wild-type cells Ras2 signals to increase protein synthesis accuracy. Other signalling pathways have much less effect on translational accuracy, as deletions of *TOR1*, *SNF1* and *TPK1/2* did not consistently or strongly affect the measured errors. Only deletion of the casein kinase II subunit *CKA1* led to small but significant increases in the measured accuracy parameters, indicating that casein kinase signalling may contribute to increased errors. In summary, we conclude that accuracy levels in ageing cells are maintained by networks of opposing signals that originate from mitochondria as well as Ras2 and other kinases.

## 3. Discussion

The relationship between ageing and translational accuracy has been discussed in the past in a number of different contexts. An early suggestion that an ‘error catastrophe’ might underlie much of the deterioration of performance in ageing cells [33] was later qualified [34], and the ensuing discussion of this issue continued for several decades after the original hypothesis [35]. One of the cornerstones of this discussion was a repeated finding, in different systems and using different methods, that ageing cells and tissues do not display significantly different error levels from young ones [22,25,36–39]. The data in these studies were derived from chronologically aged cell lines as well as tissue samples from ageing animals, and together with the replicative ageing data presented here present strong evidence that protein synthesis accuracy does not change significantly with age in most organisms.

While the stability of error rates during ageing is now generally accepted, the underlying reasons for this stability have not been fully elucidated, as many cellular processes suffer from a decline of performance in aged cells. A possible answer was proposed based on the observed reduction in the overall volume of translation in old cells and tissues and the ensuing lower elongation speed [29]. In mammalian cells, treatment with rapamycin has been reported to reduce both the speed of translation and translational errors [29], and a causal connection between the two was suggested. However, mechanistically, it is not at all clear why slower translation would necessarily mean more accurate translation. The reason why amino acid misincorporation and stop-codon read-through occur is at least in part because the ribosome must distinguish competing decoding elements, and can do so only with finite accuracy [3,40]. The more important parameter for accuracy should, therefore, be the ratio of the competing elements, and not the speed with which they are processed, especially as slower translation probably does not entail a change in the fundamental rate constants of the biochemical reactions underlying tRNA sampling by the ribosome. Our experiments where we slow down translation by various means, including control of cell division rates, depletion of translation factors and application of drugs that reduce the speed of translation, confirm that there is no uniform response of error rates to reduced translational speed, but rather that this response depends on the exact context of the experiment.

A separate question from whether error rates are affected by ageing is whether ageing is affected by altered error rates. A number of published observations indicate that this is generally the case in eukaryotic organisms, including the observation that increased transcriptional errors accelerate loss of viability during ageing [20], and that cell lines derived from species which are unusually long-lived compared with their close relatives, such as the naked mole rat, have reduced basal error rates [4]. A recent study looking at the effect of reduced accuracy resulting from impaired ribosomal methylation [19] found the opposite effect, that reduced accuracy resulted in longer lifespans. The particular response of these mutants may be a result of moderately upregulated stress response pathways [19] paired with smaller increases in errors (electronic supplementary material, figure S1), as increased activity in stress response pathways can extend lifespan in its own right [41]. By contrast, our data show that larger increases in errors without upregulated stress response pathways (figure 1) clearly shorten lifespan. We trace the molecular reasons for the interaction between translational accuracy and lifespan to an over-taxed chaperone system (figure 3), which under high error rates has to cope with the reduced folding competence arising from inaccurate translation as well as chemical damage and other age-related effects. Together, these observations may explain why it is important for ageing cells to keep error rates under tight control.

Lastly, our data show that multiple signals in the cell regulate translational accuracy in opposite ways. One of these signals consists of ROS generated in response to reduced mitochondrial function (figure 7), and this signals to increase translational errors. We do not know at the moment why cells would generate signals to decrease translational accuracy. A possible scenario is that this is an unintended by-product of the regulation of other processes, such as tRNA biogenesis. According to a recent report, ROS activate the tRNAse activity of *RNY1* [42], and altered composition of the tRNA pool could be one explanation that connects ROS and altered error levels. ROS appear to be a generally beneficial signal for healthy ageing in yeast, but the reduction in accuracy resulting from their presence must clearly be balanced by other signals to prevent negative effects on lifespan. Although we do not know the actual effectors that mediate this balance in ageing cells, our experiments reveal candidate kinase pathways (figure 8). The most prominent of these is Ras, as deletion of the *RAS2* gene has a strong negative effect on accuracy, indicating that Ras signals towards increased translational accuracy. Other pathways may further modulate the balance between pro- and anti-accuracy signals, including the Tor pathway, which was previously implicated in signalling towards reduced translation as rapamycin caused a reduction in observed error levels in mammalian cell lines. Ribosomes are targets for extensive phosphorylation [43], and it has been previously shown that such phosphorylation can affect translational accuracy [15]. Thus, we anticipate that many of the effects on translational accuracy observed upon manipulation of kinase pathways are mediated by alterations in ribosomal protein modifications, although the exact nature of the targets mediating the effects we observe remain to be elucidated.

In summary, our study reveals translational accuracy as an important parameter in ageing yeast cells, and indicates that this parameter is under active control by the cell. The ultimate aim of this control is to support functioning of the proteome under the suboptimal conditions of the ageing cell.

## 4. Material and methods

### 4.1. Strains and plasmids

All yeast strains are from the systematic genome-wide deletion collection, except for strains listed in table 1. To generate strains containing mutant alleles of the *RPS2* gene, DNA comprising this gene plus 500 nt upstream and 266 nt downstream of the gene was amplified by PCR, using primers GCGCGCGGATCCTGGCTTATTCACTAAGGATTCTTAAGGTTTTC (forward) and GCGCGCCTGCAGTAAAATTTTGATCTATTGTAGTCGCCTAATCTTGC (reverse). Genomic DNA from strain BY4741 was used as template to amplify wild-type *RPS2*, which was then cloned into pRS315 [48]. To amplify mutant alleles of *RPS2*, we obtained four strains described as *SUP38* from the National BioResource Project (NBRP) of the MEXT, Japan (http://yeast.lab.nig.ac.jp/nig/index_en.html; accession numbers BY21049, BY21050, BY21052 and BY21053). Yeast *SUP38* is known to be allelic with *SUP44/RPS2* [49]. Genomic DNA from these strains served as template for PCRs using the same primers as for the wild-type allele, and the PCR products were again cloned into pRS315. Sequencing of the cloned genes revealed that these alleles contained a Y143C mutation (*SUP38-5*) or L148S mutations (*SUP38-8* and *SUP38-9*). The fourth strain, described as *SUP38-4*, did not yield any PCR products with the *RPS2* primers. Microscopic examination of this strain showed that the cells are much smaller than typical *Saccharomyces cerevisiae* cells and we assume that this strain was mis-annotated as baker's yeast.

**Table 1.** Yeast strains used in this study.

name	genotype	reference
BY4741	Mat**a** *his3*Δ*0 leu2*Δ*0 met15*Δ*0 ura3*Δ*0*	[44]
BY4741 Δ*hsp104*	Mat**a** *his3*Δ*0 leu2*Δ*0 met15*Δ*0 ura3*Δ*0 hsp104::KanMX4*	[45]
BY4741 Δ*ssa1*	Mat**a** *his3*Δ*0 leu2*Δ*0 met15*Δ*0 ura3*Δ*0 ssa1::KanMX4*	[45]
BY4741 Δ*ssa4*	Mat**a** *his3*Δ*0 leu2*Δ*0 met15*Δ*0 ura3*Δ*0 ssa4::KanMX4*	[45]
BY4741 Δ*cox4*	Mat**a** *his3*Δ*0 leu2*Δ*0 met15*Δ*0 ura3*Δ*0 cox4::HIS3*	[32]
CGY769a	Matα *his3*Δ*200 ura3-52 leu2-3,112 tub2-201 can1-1 ACT1::HIS3*	[46]
CGY339	Matα *his3*Δ*200 ura3-52 leu2-3,112 tub2-201 ade4 act1-159::HIS3*	[46]
CGY371	MAT**a** *ura3-52 leu2 his4-539 ras2::LEU2*	[47]
CGY372	MAT**a** *ura3-52 leu2 his4-539 RAS2^ala18val19^::LEU2*	[47]
YTH222	*leu2-3,112 trp1-1 can1-100 ura3-1 ade2-1 his3-11,15 rps2::HIS3* [*URA3 RPS2*]	[15]
BY21050	Mat**a** *his5-2 leu2-1 lys1-1 met8-1 trp5-48 ura4-1 can1-100 SUP38-5*	NBRP, Japan
BY21052	Mat**a** *his5-2 leu2-1 lys1-1 met8-1 trp5-48 ura4-1 can1-100 SUP38-8*	NBRP, Japan

The wild-type and mutant plasmids were shuffled into a previously described *RPS2* shuffling strain [15] using a standard plasmid shuffling strategy [50].

Dual luciferase reporter plasmids for measuring stop-codon read-through and histidine misincorporation on CGC codons were as described [2]. The reporter for measuring lysine misincorporation on AGG codons was from Kramer *et al.* [3]. Luciferase refolding assays were conducted as described [21]. Western blots were conducted as described [51]. Storage carbohydrates were assessed using an iodine vapour assay as previously described [52].

### 4.2. Media

Yeast cells were grown either in complex medium (YPD; 1% yeast extract, 1% peptone, 2% glucose) or in synthetic complete medium (SC; 0.67% yeast nitrogen base without amino acids, 2% glucose and Kaiser dropout mixture as directed by the manufacturer (Formedium, UK)). Paromomycin was supplemented to SC to a final concentration of 0.5 g l^−1^. Glucosamine was supplemented to final concentrations from 0.2 to 6% as indicated.

### 4.3. Differential counterflow elutriation

Yeast cells were transformed with appropriate luciferase reporter constructs and grown to mid-log phase before being subjected to separation by elutriation as previously described [27]. Isolated fractions were assessed for cell size using a Casy TT cell counter (Scharfe Systems) and stained for bud scar number using FITC labelled wheat germ agglutinin (Molecular Probes).

### 4.4. Dual luciferase assays

Dual luciferase assays were conducted in 96-well microtitre plate format, using reagents from the Dual Glo luciferase assay system (Promega, UK) as described previously [53]. Growth plates used to conduct the assays were visually inspected for contaminated wells or wells with abnormal growth, and respective data were disregarded for data anlyses. For the glucosamine experiment in figure 5 only, outliers were in addition determined based on Cook's distance (more than threefold average distance) and outlier data were disregarded for data anlayses.

### 4.5. Luciferase refolding assays

Luciferase refolding assays were performed as described [21], except that heat shock was applied for 12 min, followed by addition of cyclohexmimide and further heat shock for another 12 min.

### 4.6. Ageing assays

Cultures were inoculated to starting ODs of 0.1–0.2 in 5 ml medium in sterile 50 ml plastic tubes, and incubated in a shaker at 30°C. To assess the proportion of live cells, 100 µl of cells were mixed with 10 µl of 20 µM phloxine B (Sigma-Aldrich, UK) and water in 1 ml final volume, and left for 30 min at room temperature. Stained and unstained cells were then counted manually in a haemocytometer.

### 4.7. Heat shock survival assays

Yeast cultures were inoculated from overnight cultures into YPD or minimal medium to a starting OD_600_ of 0.1, and grown to a final OD_600_ of 1–2. Cell density was determined using a haemocytometer, and cells were diluted into 1 ml of fresh medium to a density of 5000 cells ml^−1^. Three 100 µl portions of this diluted culture were plated onto three YPD plates to determine pre-heat shock CFU densities. The remaining 700 µl of culture were incubated in a 49°C water bath for 10 min, followed by transfer to a 20°C water bath for 5 min. Three further 100 µl portions were then plated onto YPD plates to determine post-heat-shock CFU densities. Plates were incubated for 48 h and colonies counted.

### 4.8 Fluoresence microscopy

Cells were viewed with an Olympus IX-81 fluorescence microscope with a 150 W xenon/mercury lamp and an Olympus 150× Plan NeoFluor oil-immersion objective. Images were captured using a Hammamatsu ORCA AG digital camera using Olympus Cell R software. Bud scars were visualized after incubation with 5 µg ml^−1^ FITC-Wheat Germ agglutinin in PBS for 10 min.

### 4.9. Statistical analyses

All statistical analyses were conducted in R v. 3.2.3 [54]. Except for figure 8*a*, data were analysed using one- or two-way analyses of variance as appropriate, with Tukey's HSD as a *post hoc* test. Data in figure 8*a* were analysed using the Kruskal–Wallis test, with Nemenyi's test as implemented in the PMCMR package as *post hoc* test. Statistical significance is indicated in all figures with the following symbols: no symbol or n.s., *p* > 0.05; *, 0.05 > *p* > 0.01; **, 0.01 > *p* > 0.001; ***, 0.001 > *p*.

## Supplementary Material

Supplemental Figure 1

## Supplementary Material

Raw data files

## Data accessibility

Raw data for this study (growth assays, ageing assays and dual luciferase assays) are provided in the electronic supplementary material, S2.
