# Supplementary material for: The control of translational accuracy is a determinant of healthy ageing in yeast
Source: Open Biol. 2017 Jan 18;7(1):160291. doi: 10.1098/rsob.160291 (PMC5303280; doi:10.1098/rsob.160291)
Supplement: Supplemental Figure 1 [file rsob160291supp1.pdf]

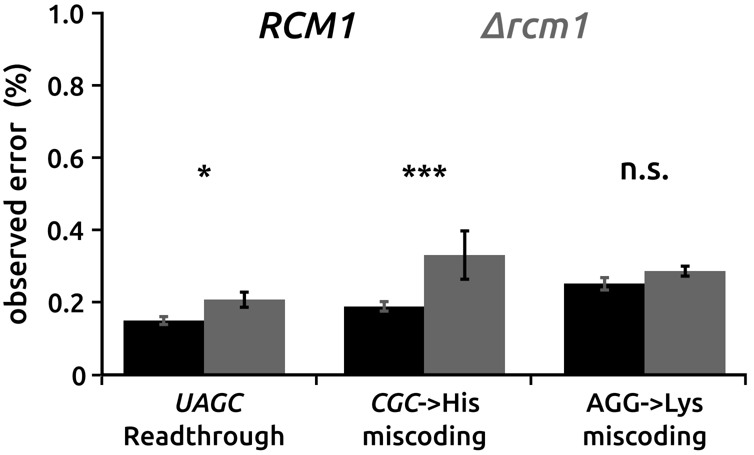

Supplemental Figure 1. Translational accuracy in an *rcm1* deletion strain. Bars indicate averages and standard deviations obtained with eight separate transformants. Statistical significance as determined by one-way ANOVA and post-hoc testing (Tukey HSD) is indicated as follows: n.s., $p>0.05$; *, $p<0.05$; ***, $p<0.001$.
